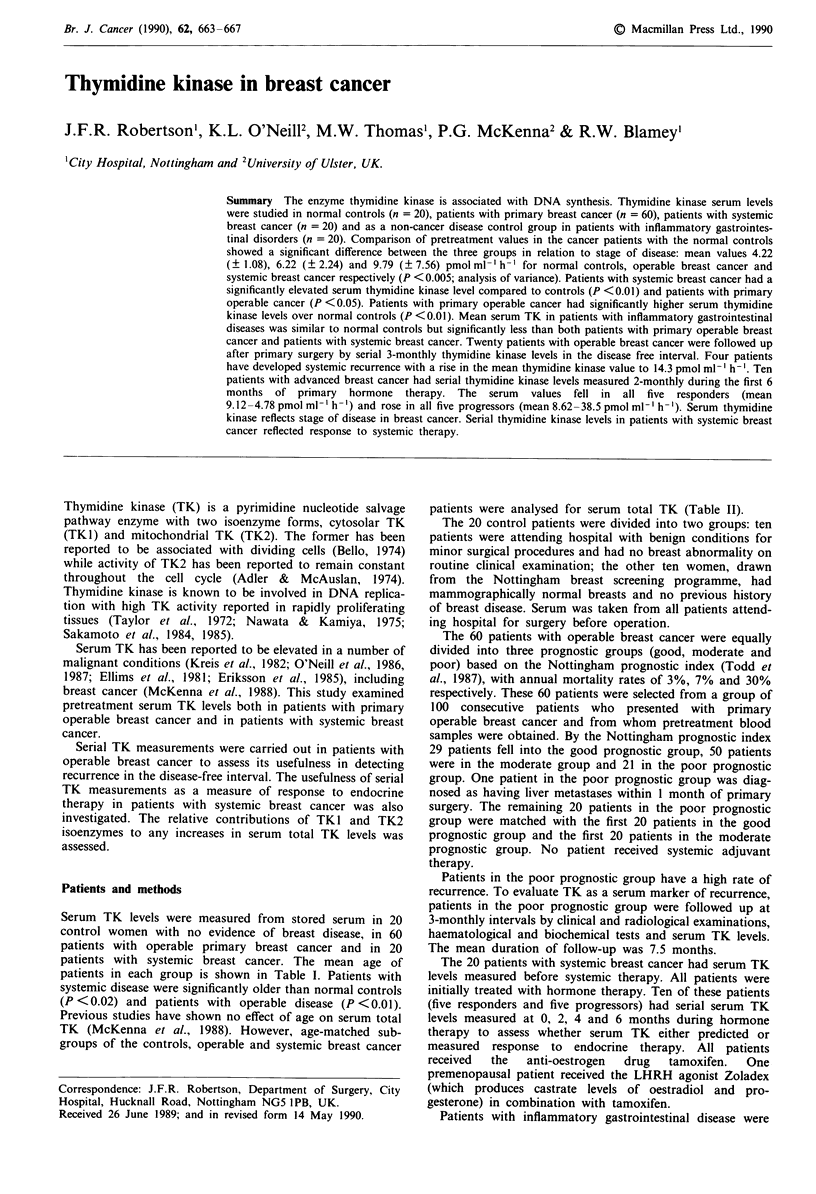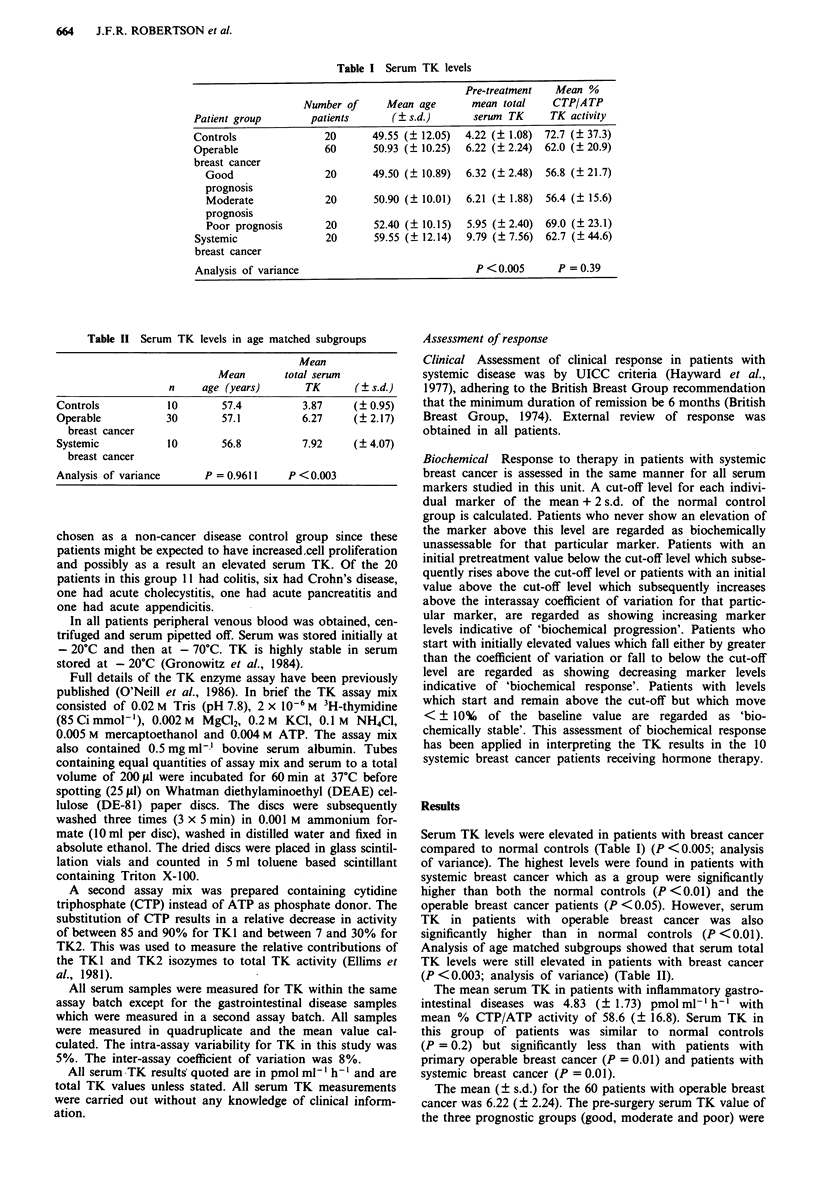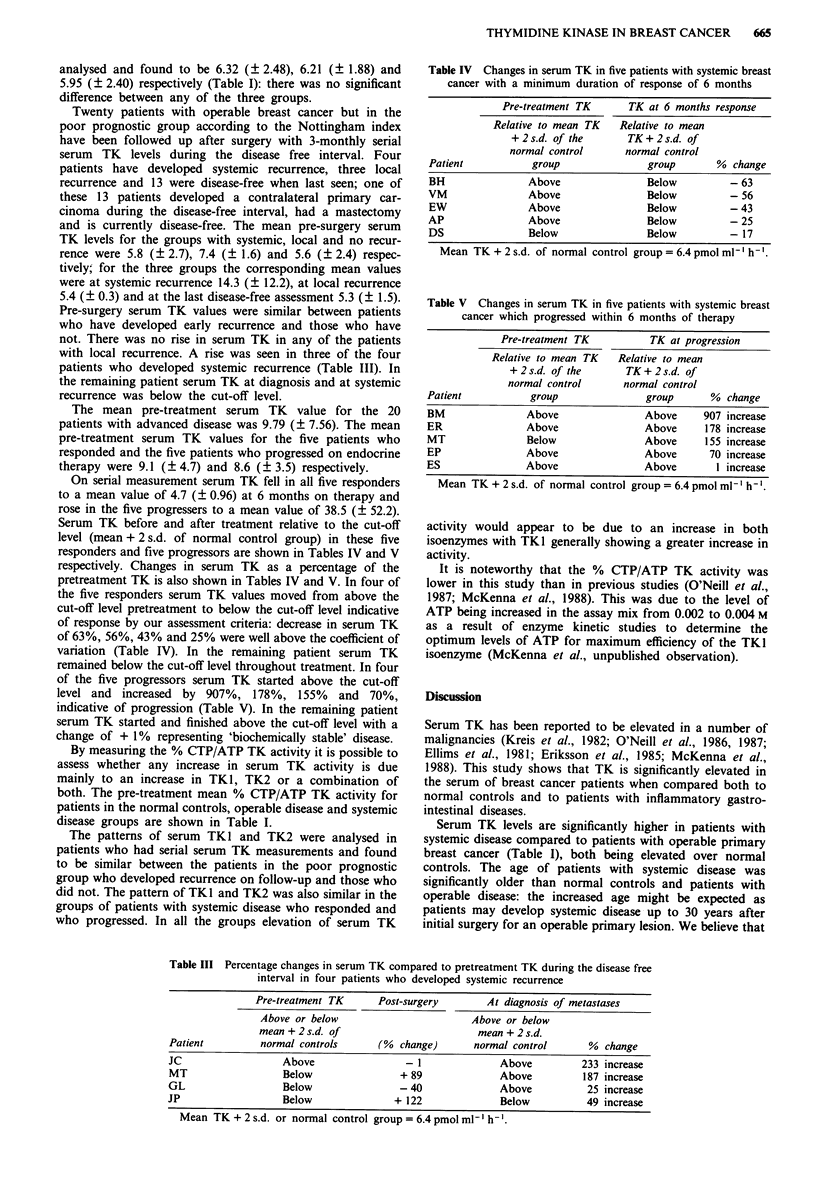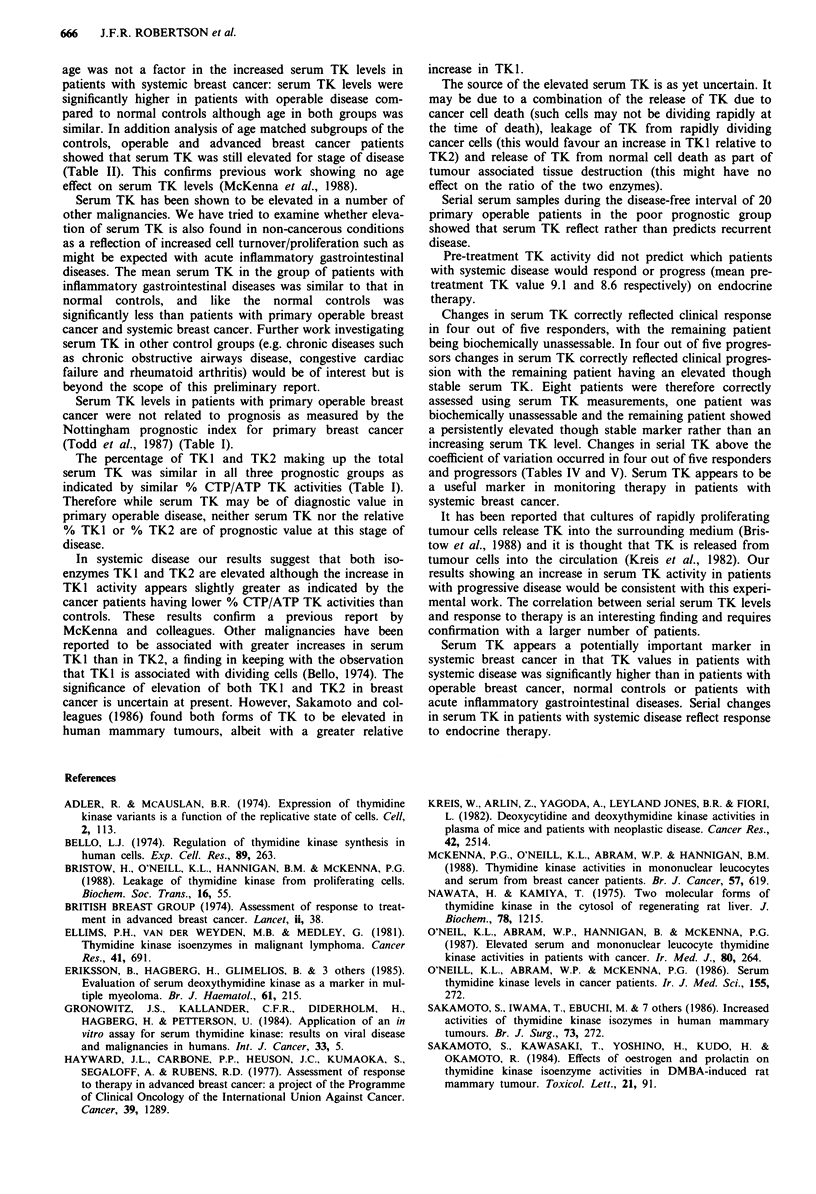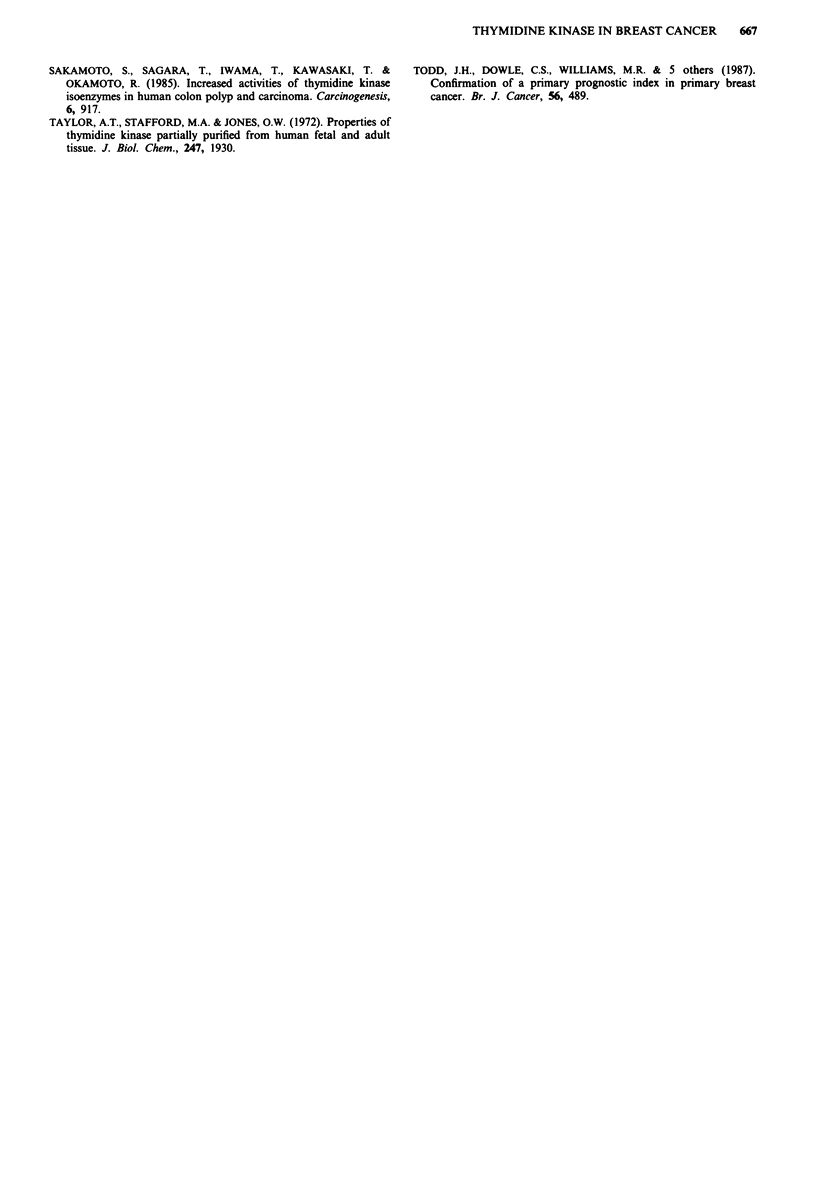# Thymidine kinase in breast cancer.

**DOI:** 10.1038/bjc.1990.352

**Published:** 1990-10

**Authors:** J. F. Robertson, K. L. O'Neill, M. W. Thomas, P. G. McKenna, R. W. Blamey

**Affiliations:** City Hospital, Nottingham, UK.

## Abstract

The enzyme thymidine kinase is associated with DNA synthesis. Thymidine kinase serum levels were studied in normal controls (n = 20), patients with primary breast cancer (n = 60), patients with systemic breast cancer (n = 20) and as a non-cancer disease control group in patients with inflammatory gastrointestinal disorders (n = 20). Comparison of pretreatment values in the cancer patients with the normal controls showed a significant difference between the three groups in relation to stage of disease: mean values 4.22 (+/- 1.08), 6.22 (+/- 2.24) and 9.79 (+/- 7.56) pmol ml-1 h-1 for normal controls, operable breast cancer and systemic breast cancer respectively (P less than 0.005; analysis of variance). Patients with systemic breast cancer had a significantly elevated serum thymidine kinase level compared to controls (P less than 0.01) and patients with primary operable cancer (P less than 0.05). Patients with primary operable cancer had significantly higher serum thymidine kinase levels over normal controls (P less than 0.01). Mean serum TK in patients with inflammatory gastrointestinal diseases was similar to normal controls but significantly less than both patients with primary operable breast cancer and patients with systemic breast cancer. Twenty patients with operable breast cancer were followed up after primary surgery by serial 3-monthly thymidine kinase levels in the disease free interval. Four patients have developed systemic recurrence with a rise in the mean thymidine kinase value to 14.3 pmol ml-1 h-1. Ten patients with advanced breast cancer had serial thymidine kinase levels measured 2-monthly during the first 6 months of primary hormone therapy. The serum values fell in all five responders (mean 9.12-4.78 pmol ml-1 h-1) and rose in all five progressors (mean 8.62-38.5 pmol ml-1 h-1). Serum thymidine kinase reflects stage of disease in breast cancer. Serial thymidine kinase levels in patients with systemic breast cancer reflected response to systemic therapy.


Br. J. Cancer (1990), 62, 663-667 © Macmillan Press Ltd., 1990

### Thymidine kinase in breast cancer

J.F.R. Robertson[1], K.L. O'Neill[2], M.W. Thomas[1], P.G. McKenna[2] & R.W. Blamey[1]

[1]City Hospital, Nottingham and [2]University of Ulster, UK.

**Summary** The enzyme thymidine kinase is associated with DNA synthesis. Thymidine kinase serum levels were studied in normal controls ($n$ = 20), patients with primary breast cancer ($n$ = 60), patients with systemic breast cancer ($n$ = 20) and as a non-cancer disease control group in patients with inflammatory gastrointestinal disorders ($n$ = 20). Comparison of pretreatment values in the cancer patients with the normal controls showed a significant difference between the three groups in relation to stage of disease: mean values 4.22 (± 1.08), 6.22 (± 2.24) and 9.79 (± 7.56) pmol $ml^{-1}$ $h^{-1}$ for normal controls, operable breast cancer and systemic breast cancer respectively ($P$ <0.005; analysis of variance). Patients with systemic breast cancer had a significantly elevated serum thymidine kinase level compared to controls ($P$ <0.01) and patients with primary operable cancer ($P$ <0.05). Patients with primary operable cancer had significantly higher serum thymidine kinase levels over normal controls ($P$ <0.01). Mean serum TK in patients with inflammatory gastrointestinal diseases was similar to normal controls but significantly less than both patients with primary operable breast cancer and patients with systemic breast cancer. Twenty patients with operable breast cancer were followed up after primary surgery by serial 3-monthly thymidine kinase levels in the disease free interval. Four patients have developed systemic recurrence with a rise in the mean thymidine kinase value to 14.3 pmol $ml^{-1}$ $h^{-1}$. Ten patients with advanced breast cancer had serial thymidine kinase levels measured 2-monthly during the first 6 months of primary hormone therapy. The serum values fell in all five responders (mean 9.12-4.78 pmol $ml^{-1}$ $h^{-1}$) and rose in all five progressors (mean 8.62-38.5 pmol $ml^{-1}$ $h^{-1}$). Serum thymidine kinase reflects stage of disease in breast cancer. Serial thymidine kinase levels in patients with systemic breast cancer reflected response to systemic therapy.

Thymidine kinase (TK) is a pyrimidine nucleotide salvage pathway enzyme with two isoenzyme forms, cytosolar TK (TK1) and mitochondrial TK (TK2). The former has been reported to be associated with dividing cells (Bello, 1974) while activity of TK2 has been reported to remain constant throughout the cell cycle (Adler & McAuslan, 1974). Thymidine kinase is known to be involved in DNA replication with high TK activity reported in rapidly proliferating tissues (Taylor *et al.*, 1972; Nawata & Kamiya, 1975; Sakamoto *et al.*, 1984, 1985).

Serum TK has been reported to be elevated in a number of malignant conditions (Kreis *et al.*, 1982; O'Neill *et al.*, 1986, 1987; Ellims *et al.*, 1981; Eriksson *et al.*, 1985), including breast cancer (McKenna *et al.*, 1988). This study examined pretreatment serum TK levels both in patients with primary operable breast cancer and in patients with systemic breast cancer.

Serial TK measurements were carried out in patients with operable breast cancer to assess its usefulness in detecting recurrence in the disease-free interval. The usefulness of serial TK measurements as a measure of response to endocrine therapy in patients with systemic breast cancer was also investigated. The relative contributions of TK1 and TK2 isoenzymes to any increases in serum total TK levels was assessed.

### Patients and methods

Serum TK levels were measured from stored serum in 20 control women with no evidence of breast disease, in 60 patients with operable primary breast cancer and in 20 patients with systemic breast cancer. The mean age of patients in each group is shown in Table I. Patients with systemic disease were significantly older than normal controls ($P$ <0.02) and patients with operable disease ($P$ <0.01). Previous studies have shown no effect of age on serum total TK (McKenna *et al.*, 1988). However, age-matched subgroups of the controls, operable and systemic breast cancer patients were analysed for serum total TK (Table II).

The 20 control patients were divided into two groups: ten patients were attending hospital with benign conditions for minor surgical procedures and had no breast abnormality on routine clinical examination; the other ten women, drawn from the Nottingham breast screening programme, had mammographically normal breasts and no previous history of breast disease. Serum was taken from all patients attending hospital for surgery before operation.

The 60 patients with operable breast cancer were equally divided into three prognostic groups (good, moderate and poor) based on the Nottingham prognostic index (Todd *et al.*, 1987), with annual mortality rates of 3%, 7% and 30% respectively. These 60 patients were selected from a group of 100 consecutive patients who presented with primary operable breast cancer and from whom pretreatment blood samples were obtained. By the Nottingham prognostic index 29 patients fell into the good prognostic group, 50 patients were in the moderate group and 21 in the poor prognostic group. One patient in the poor prognostic group was diagnosed as having liver metastases within 1 month of primary surgery. The remaining 20 patients in the poor prognostic group were matched with the first 20 patients in the good prognostic group and the first 20 patients in the moderate prognostic group. No patient received systemic adjuvant therapy.

Patients in the poor prognostic group have a high rate of recurrence. To evaluate TK as a serum marker of recurrence, patients in the poor prognostic group were followed up at 3-monthly intervals by clinical and radiological examinations, haematological and biochemical tests and serum TK levels. The mean duration of follow-up was 7.5 months.

The 20 patients with systemic breast cancer had serum TK levels measured before systemic therapy. All patients were initially treated with hormone therapy. Ten of these patients (five responders and five progressors) had serial serum TK levels measured at 0, 2, 4 and 6 months during hormone therapy to assess whether serum TK either predicted or measured response to endocrine therapy. All patients received the anti-oestrogen drug tamoxifen. One premenopausal patient received the LHRH agonist Zoladex (which produces castrate levels of oestradiol and progesterone) in combination with tamoxifen.

Patients with inflammatory gastrointestinal disease were

Correspondence: J.F.R. Robertson, Department of Surgery, City Hospital, Hucknall Road, Nottingham NG5 1PB, UK.
Received 26 June 1989; and in revised form 14 May 1990.

**Table I** Serum TK levels

| Patient group | Number of patients | Mean age (± s.d.) | Pre-treatment mean total serum TK | Mean % CTP/ATP TK activity |
|---|---|---|---|---|
| Controls | 20 | 49.55 (± 12.05) | 4.22 (± 1.08) | 72.7 (± 37.3) |
| Operable breast cancer | 60 | 50.93 (± 10.25) | 6.22 (± 2.24) | 62.0 (± 20.9) |
| Good prognosis | 20 | 49.50 (± 10.89) | 6.32 (± 2.48) | 56.8 (± 21.7) |
| Moderate prognosis | 20 | 50.90 (± 10.01) | 6.21 (± 1.88) | 56.4 (± 15.6) |
| Poor prognosis | 20 | 52.40 (± 10.15) | 5.95 (± 2.40) | 69.0 (± 23.1) |
| Systemic breast cancer | 20 | 59.55 (± 12.14) | 9.79 (± 7.56) | 62.7 (± 44.6) |
| Analysis of variance | | | $P < 0.005$ | $P = 0.39$ |

**Table II** Serum TK levels in age matched subgroups

| | n | Mean age (years) | Mean total serum TK | (± s.d.) |
|---|---|---|---|---|
| Controls | 10 | 57.4 | 3.87 | (± 0.95) |
| Operable breast cancer | 30 | 57.1 | 6.27 | (± 2.17) |
| Systemic breast cancer | 10 | 56.8 | 7.92 | (± 4.07) |
| Analysis of variance | | $P = 0.9611$ | $P < 0.003$ | |

chosen as a non-cancer disease control group since these patients might be expected to have increased cell proliferation and possibly as a result an elevated serum TK. Of the 20 patients in this group 11 had colitis, six had Crohn's disease, one had acute cholecystitis, one had acute pancreatitis and one had acute appendicitis.

In all patients peripheral venous blood was obtained, centrifuged and serum pipetted off. Serum was stored initially at − 20°C and then at − 70°C. TK is highly stable in serum stored at − 20°C (Gronowitz et al., 1984).

Full details of the TK enzyme assay have been previously published (O'Neill et al., 1986). In brief the TK assay mix consisted of 0.02 M Tris (pH 7.8), $2 \times 10^{-6}$ M $^3$H-thymidine (85 Ci mmol$^{-1}$), 0.002 M $MgCl_2$, 0.2 M KCl, 0.1 M $NH_4Cl$, 0.005 M mercaptoethanol and 0.004 M ATP. The assay mix also contained 0.5 mg ml$^{-1}$ bovine serum albumin. Tubes containing equal quantities of assay mix and serum to a total volume of 200 µl were incubated for 60 min at 37°C before spotting (25 µl) on Whatman diethylaminoethyl (DEAE) cellulose (DE-81) paper discs. The discs were subsequently washed three times (3 × 5 min) in 0.001 M ammonium formate (10 ml per disc), washed in distilled water and fixed in absolute ethanol. The dried discs were placed in glass scintillation vials and counted in 5 ml toluene based scintillant containing Triton X-100.

A second assay mix was prepared containing cytidine triphosphate (CTP) instead of ATP as phosphate donor. The substitution of CTP results in a relative decrease in activity of between 85 and 90% for TK1 and between 7 and 30% for TK2. This was used to measure the relative contributions of the TK1 and TK2 isozymes to total TK activity (Ellims et al., 1981).

All serum samples were measured for TK within the same assay batch except for the gastrointestinal disease samples which were measured in a second assay batch. All samples were measured in quadruplicate and the mean value calculated. The intra-assay variability for TK in this study was 5%. The inter-assay coefficient of variation was 8%.

All serum TK results quoted are in pmol ml$^{-1}$ h$^{-1}$ and are total TK values unless stated. All serum TK measurements were carried out without any knowledge of clinical information.

*Assessment of response*

*Clinical* Assessment of clinical response in patients with systemic disease was by UICC criteria (Hayward et al., 1977), adhering to the British Breast Group recommendation that the minimum duration of remission be 6 months (British Breast Group, 1974). External review of response was obtained in all patients.

*Biochemical* Response to therapy in patients with systemic breast cancer is assessed in the same manner for all serum markers studied in this unit. A cut-off level for each individual marker of the mean + 2 s.d. of the normal control group is calculated. Patients who never show an elevation of the marker above this level are regarded as biochemically unassessable for that particular marker. Patients with an initial pretreatment value below the cut-off level which subsequently rises above the cut-off level or patients with an initial value above the cut-off level which subsequently increases above the interassay coefficient of variation for that particular marker, are regarded as showing increasing marker levels indicative of 'biochemical progression'. Patients who start with initially elevated values which fall either by greater than the coefficient of variation or fall to below the cut-off level are regarded as showing decreasing marker levels indicative of 'biochemical response'. Patients with levels which start and remain above the cut-off but which move $< \pm 10\%$ of the baseline value are regarded as 'biochemically stable'. This assessment of biochemical response has been applied in interpreting the TK results in the 10 systemic breast cancer patients receiving hormone therapy.

### Results

Serum TK levels were elevated in patients with breast cancer compared to normal controls (Table I) ($P < 0.005$; analysis of variance). The highest levels were found in patients with systemic breast cancer which as a group were significantly higher than both the normal controls ($P < 0.01$) and the operable breast cancer patients ($P < 0.05$). However, serum TK in patients with operable breast cancer was also significantly higher than in normal controls ($P < 0.01$). Analysis of age matched subgroups showed that serum total TK levels were still elevated in patients with breast cancer ($P < 0.003$; analysis of variance) (Table II).

The mean serum TK in patients with inflammatory gastrointestinal diseases was 4.83 (± 1.73) pmol ml$^{-1}$ h$^{-1}$ with mean % CTP/ATP activity of 58.6 (± 16.8). Serum TK in this group of patients was similar to normal controls ($P = 0.2$) but significantly less than with patients with primary operable breast cancer ($P = 0.01$) and patients with systemic breast cancer ($P = 0.01$).

The mean (± s.d.) for the 60 patients with operable breast cancer was 6.22 (± 2.24). The pre-surgery serum TK value of the three prognostic groups (good, moderate and poor) were analysed and found to be 6.32 (± 2.48), 6.21 (± 1.88) and 5.95 (± 2.40) respectively (Table I): there was no significant difference between any of the three groups.

Twenty patients with operable breast cancer but in the poor prognostic group according to the Nottingham index have been followed up after surgery with 3-monthly serial serum TK levels during the disease free interval. Four patients have developed systemic recurrence, three local recurrence and 13 were disease-free when last seen; one of these 13 patients developed a contralateral primary carcinoma during the disease-free interval, had a mastectomy and is currently disease-free. The mean pre-surgery serum TK levels for the groups with systemic, local and no recurrence were 5.8 (± 2.7), 7.4 (± 1.6) and 5.6 (± 2.4) respectively; for the three groups the corresponding mean values were at systemic recurrence 14.3 (± 12.2), at local recurrence 5.4 (± 0.3) and at the last disease-free assessment 5.3 (± 1.5). Pre-surgery serum TK values were similar between patients who have developed early recurrence and those who have not. There was no rise in serum TK in any of the patients with local recurrence. A rise was seen in three of the four patients who developed systemic recurrence (Table III). In the remaining patient serum TK at diagnosis and at systemic recurrence was below the cut-off level.

The mean pre-treatment serum TK value for the 20 patients with advanced disease was 9.79 (± 7.56). The mean pre-treatment serum TK values for the five patients who responded and the five patients who progressed on endocrine therapy were 9.1 (± 4.7) and 8.6 (± 3.5) respectively.

On serial measurement serum TK fell in all five responders to a mean value of 4.7 (± 0.96) at 6 months on therapy and rose in the five progressers to a mean value of 38.5 (± 52.2). Serum TK before and after treatment relative to the cut-off level (mean + 2 s.d. of normal control group) in these five responders and five progressors are shown in Tables IV and V respectively. Changes in serum TK as a percentage of the pretreatment TK is also shown in Tables IV and V. In four of the five responders serum TK values moved from above the cut-off level pretreatment to below the cut-off level indicative of response by our assessment criteria: decrease in serum TK of 63%, 56%, 43% and 25% were well above the coefficient of variation (Table IV). In the remaining patient serum TK remained below the cut-off level throughout treatment. In four of the five progressors serum TK started above the cut-off level and increased by 907%, 178%, 155% and 70%, indicative of progression (Table V). In the remaining patient serum TK started and finished above the cut-off level with a change of + 1% representing 'biochemically stable' disease.

By measuring the % CTP/ATP TK activity it is possible to assess whether any increase in serum TK activity is due mainly to an increase in TK1, TK2 or a combination of both. The pre-treatment mean % CTP/ATP TK activity for patients in the normal controls, operable disease and systemic disease groups are shown in Table I.

The patterns of serum TK1 and TK2 were analysed in patients who had serial serum TK measurements and found to be similar between the patients in the poor prognostic group who developed recurrence on follow-up and those who did not. The pattern of TK1 and TK2 was also similar in the groups of patients with systemic disease who responded and who progressed. In all the groups elevation of serum TK activity would appear to be due to an increase in both isoenzymes with TK1 generally showing a greater increase in activity.

It is noteworthy that the % CTP/ATP TK activity was lower in this study than in previous studies (O'Neill *et al.*, 1987; McKenna *et al.*, 1988). This was due to the level of ATP being increased in the assay mix from 0.002 to 0.004 M as a result of enzyme kinetic studies to determine the optimum levels of ATP for maximum efficiency of the TK1 isoenzyme (McKenna *et al.*, unpublished observation).

**Table IV** Changes in serum TK in five patients with systemic breast cancer with a minimum duration of response of 6 months

| Patient | Pre-treatment TK: Relative to mean TK + 2 s.d. of the normal control group | TK at 6 months response: Relative to mean TK + 2 s.d. of normal control group | % change |
|---|---|---|---|
| BH | Above | Below | − 63 |
| VM | Above | Below | − 56 |
| EW | Above | Below | − 43 |
| AP | Above | Below | − 25 |
| DS | Below | Below | − 17 |

Mean TK + 2 s.d. of normal control group = 6.4 pmol $ml^{-1}$ $h^{-1}$.

**Table V** Changes in serum TK in five patients with systemic breast cancer which progressed within 6 months of therapy

| Patient | Pre-treatment TK: Relative to mean TK + 2 s.d. of the normal control group | TK at progression: Relative to mean TK + 2 s.d. of normal control group | % change |
|---|---|---|---|
| BM | Above | Above | 907 increase |
| ER | Above | Above | 178 increase |
| MT | Below | Above | 155 increase |
| EP | Above | Above | 70 increase |
| ES | Above | Above | 1 increase |

Mean TK + 2 s.d. of normal control group = 6.4 pmol $ml^{-1}$ $h^{-1}$.

### Discussion

Serum TK has been reported to be elevated in a number of malignancies (Kreis *et al.*, 1982; O'Neill *et al.*, 1986, 1987; Ellims *et al.*, 1981; Eriksson *et al.*, 1985; McKenna *et al.*, 1988). This study shows that TK is significantly elevated in the serum of breast cancer patients when compared both to normal controls and to patients with inflammatory gastrointestinal diseases.

Serum TK levels are significantly higher in patients with systemic disease compared to patients with operable primary breast cancer (Table I), both being elevated over normal controls. The age of patients with systemic disease was significantly older than normal controls and patients with operable disease: the increased age might be expected as patients may develop systemic disease up to 30 years after initial surgery for an operable primary lesion. We believe that

**Table III** Percentage changes in serum TK compared to pretreatment TK during the disease free interval in four patients who developed systemic recurrence

| Patient | Pre-treatment TK: Above or below mean + 2 s.d. of normal controls | Post-surgery (% change) | At diagnosis of metastases: Above or below mean + 2 s.d. normal control | % change |
|---|---|---|---|---|
| JC | Above | − 1 | Above | 233 increase |
| MT | Below | + 89 | Above | 187 increase |
| GL | Below | − 40 | Above | 25 increase |
| JP | Below | + 122 | Below | 49 increase |

Mean TK + 2 s.d. or normal control group = 6.4 pmol $ml^{-1}$ $h^{-1}$.

age was not a factor in the increased serum TK levels in patients with systemic breast cancer: serum TK levels were significantly higher in patients with operable disease compared to normal controls although age in both groups was similar. In addition analysis of age matched subgroups of the controls, operable and advanced breast cancer patients showed that serum TK was still elevated for stage of disease (Table II). This confirms previous work showing no age effect on serum TK levels (McKenna *et al.*, 1988).

Serum TK has been shown to be elevated in a number of other malignancies. We have tried to examine whether elevation of serum TK is also found in non-cancerous conditions as a reflection of increased cell turnover/proliferation such as might be expected with acute inflammatory gastrointestinal diseases. The mean serum TK in the group of patients with inflammatory gastrointestinal diseases was similar to that in normal controls, and like the normal controls was significantly less than patients with primary operable breast cancer and systemic breast cancer. Further work investigating serum TK in other control groups (e.g. chronic diseases such as chronic obstructive airways disease, congestive cardiac failure and rheumatoid arthritis) would be of interest but is beyond the scope of this preliminary report.

Serum TK levels in patients with primary operable breast cancer were not related to prognosis as measured by the Nottingham prognostic index for primary breast cancer (Todd *et al.*, 1987) (Table I).

The percentage of TK1 and TK2 making up the total serum TK was similar in all three prognostic groups as indicated by similar % CTP/ATP TK activities (Table I). Therefore while serum TK may be of diagnostic value in primary operable disease, neither serum TK nor the relative % TK1 or % TK2 are of prognostic value at this stage of disease.

In systemic disease our results suggest that both isoenzymes TK1 and TK2 are elevated although the increase in TK1 activity appears slightly greater as indicated by the cancer patients having lower % CTP/ATP TK activities than controls. These results confirm a previous report by McKenna and colleagues. Other malignancies have been reported to be associated with greater increases in serum TK1 than in TK2, a finding in keeping with the observation that TK1 is associated with dividing cells (Bello, 1974). The significance of elevation of both TK1 and TK2 in breast cancer is uncertain at present. However, Sakamoto and colleagues (1986) found both forms of TK to be elevated in human mammary tumours, albeit with a greater relative increase in TK1.

The source of the elevated serum TK is as yet uncertain. It may be due to a combination of the release of TK due to cancer cell death (such cells may not be dividing rapidly at the time of death), leakage of TK from rapidly dividing cancer cells (this would favour an increase in TK1 relative to TK2) and release of TK from normal cell death as part of tumour associated tissue destruction (this might have no effect on the ratio of the two enzymes).

Serial serum samples during the disease-free interval of 20 primary operable patients in the poor prognostic group showed that serum TK reflect rather than predicts recurrent disease.

Pre-treatment TK activity did not predict which patients with systemic disease would respond or progress (mean pre-treatment TK value 9.1 and 8.6 respectively) on endocrine therapy.

Changes in serum TK correctly reflected clinical response in four out of five responders, with the remaining patient being biochemically unassessable. In four out of five progressors changes in serum TK correctly reflected clinical progression with the remaining patient having an elevated though stable serum TK. Eight patients were therefore correctly assessed using serum TK measurements, one patient was biochemically unassessable and the remaining patient showed a persistently elevated though stable marker rather than an increasing serum TK level. Changes in serial TK above the coefficient of variation occurred in four out of five responders and progressors (Tables IV and V). Serum TK appears to be a useful marker in monitoring therapy in patients with systemic breast cancer.

It has been reported that cultures of rapidly proliferating tumour cells release TK into the surrounding medium (Bristow *et al.*, 1988) and it is thought that TK is released from tumour cells into the circulation (Kreis *et al.*, 1982). Our results showing an increase in serum TK activity in patients with progressive disease would be consistent with this experimental work. The correlation between serial serum TK levels and response to therapy is an interesting finding and requires confirmation with a larger number of patients.

Serum TK appears a potentially important marker in systemic breast cancer in that TK values in patients with systemic disease was significantly higher than in patients with operable breast cancer, normal controls or patients with acute inflammatory gastrointestinal diseases. Serial changes in serum TK in patients with systemic disease reflect response to endocrine therapy.

### References

ADLER, R. & MCAUSLAN, B.R. (1974). Expression of thymidine kinase variants is a function of the replicative state of cells. *Cell*, **2**, 113.

BELLO, L.J. (1974). Regulation of thymidine kinase synthesis in human cells. *Exp. Cell. Res.*, **89**, 263.

BRISTOW, H., O'NEILL, K.L., HANNIGAN, B.M. & MCKENNA, P.G. (1988). Leakage of thymidine kinase from proliferating cells. *Biochem. Soc. Trans.*, **16**, 55.

BRITISH BREAST GROUP (1974). Assessment of response to treatment in advanced breast cancer. *Lancet*, **ii**, 38.

ELLIMS, P.H., VAN DER WEYDEN, M.B. & MEDLEY, G. (1981). Thymidine kinase isoenzymes in malignant lymphoma. *Cancer Res.*, **41**, 691.

ERIKSSON, B., HAGBERG, H., GLIMELIOS, B. & 3 others (1985). Evaluation of serum deoxythymidine kinase as a marker in multiple myeloma. *Br. J. Haematol.*, **61**, 215.

GRONOWITZ, J.S., KALLANDER, C.F.R., DIDERHOLM, H., HAGBERG, H. & PETTERSON, U. (1984). Application of an *in vitro* assay for serum thymidine kinase: results on viral disease and malignancies in humans. *Int. J. Cancer*, **33**, 5.

HAYWARD, J.L., CARBONE, P.P., HEUSON, J.C., KUMAOKA, S., SEGALOFF, A. & RUBENS, R.D. (1977). Assessment of response to therapy in advanced breast cancer: a project of the Programme of Clinical Oncology of the International Union Against Cancer. *Cancer*, **39**, 1289.

KREIS, W., ARLIN, Z., YAGODA, A., LEYLAND JONES, B.R. & FIORI, L. (1982). Deoxycytidine and deoxythymidine kinase activities in plasma of mice and patients with neoplastic disease. *Cancer Res.*, **42**, 2514.

MCKENNA, P.G., O'NEILL, K.L., ABRAM, W.P. & HANNIGAN, B.M. (1988). Thymidine kinase activities in mononuclear leucocytes and serum from breast cancer patients. *Br. J. Cancer*, **57**, 619.

NAWATA, H. & KAMIYA, T. (1975). Two molecular forms of thymidine kinase in the cytosol of regenerating rat liver. *J. Biochem.*, **78**, 1215.

O'NEIL, K.L., ABRAM, W.P., HANNIGAN, B. & MCKENNA, P.G. (1987). Elevated serum and mononuclear leucocyte thymidine kinase activities in patients with cancer. *Ir. Med. J.*, **80**, 264.

O'NEILL, K.L., ABRAM, W.P. & MCKENNA, P.G. (1986). Serum thymidine kinase levels in cancer patients. *Ir. J. Med. Sci.*, **155**, 272.

SAKAMOTO, S., IWAMA, T., EBUCHI, M. & 7 others (1986). Increased activities of thymidine kinase isozymes in human mammary tumours. *Br. J. Surg.*, **73**, 272.

SAKAMOTO, S., KAWASAKI, T., YOSHINO, H., KUDO, H. & OKAMOTO, R. (1984). Effects of oestrogen and prolactin on thymidine kinase isoenzyme activities in DMBA-induced rat mammary tumour. *Toxicol. Lett.*, **21**, 91.

SAKAMOTO, S., SAGARA, T., IWAMA, T., KAWASAKI, T. & OKAMOTO, R. (1985). Increased activities of thymidine kinase isoenzymes in human colon polyp and carcinoma. *Carcinogenesis*, **6**, 917.

TAYLOR, A.T., STAFFORD, M.A. & JONES, O.W. (1972). Properties of thymidine kinase partially purified from human fetal and adult tissue. *J. Biol. Chem.*, **247**, 1930.

TODD, J.H., DOWLE, C.S., WILLIAMS, M.R. & 5 others (1987). Confirmation of a primary prognostic index in primary breast cancer. *Br. J. Cancer*, **56**, 489.